# Effect of Boron Additions on the Microstructural Evolution and Properties of Fe-Mo-Cu-Ni-C Sintered Steel

**DOI:** 10.3390/ma16216953

**Published:** 2023-10-30

**Authors:** Zenglin Liu, Yankang Wang, Yong Yuan, Fenghua Luo, Tao Wang, Wei Han, Liming Tan

**Affiliations:** 1 China Iron and Steel Research Institute Group, Beijing 100081, China; lgliuzenglin@163.com; 2 Shandong Luyin New Material Technology Co., Ltd., Jinan 271104, China; wangyankang1211@163.com (Y.W.); lgyuanyong@163.com (Y.Y.); 18315687982@163.com (T.W.); 3 State Key Laboratory of Powder Metallurgy, Central South University, Changsha 410083, China

**Keywords:** PM sintered steel, pre-alloyed powder, boron addition, microstructure, properties

## Abstract

The effects of different boron (B) additions from 0 to 0.5 wt.% on the microstructure and properties of Fe-Mo-Cu-Ni-*x*B-C powder metallurgy (PM) steels were investigated in this work. The results indicated that the ferrite phase quantity decreased and disappeared, Ni/Cu became more homogeneous, and M_2_B phase formed, with the addition of B. The density and hardness of the sintered steels monotonously increased with increasing B content, whereas the tensile strength and impact toughness first increased and then decreased. The tensile strength of the steels reached a maximum value of 1097 MPa at a 0.2% B content, whereas the impact toughness reached a maximum value of 25.7 J/cm^2^ at a 0.1% B content and then sharply decreased when the B content exceeded 0.2%. Frictional wear experiments showed that the weight loss of the steels decreased with an increasing B content under low load conditions (100 N), and the lowest weight loss of 0.043 g occurred at a 0.2% B content. Under high load conditions (200 N), the 0.1% B content steel saw the lowest weight loss 0.075 g, exhibiting excellent wear resistance, but the abrasive resistance of the steels decreased with a further increase in the B content due to the germination of microcracks and large spalling caused by the high hardness and brittleness.

## 1. Introduction

Powder metallurgy (PM) is a versatile method for mass producing nearly net-shaped parts with low cost, accurate and reproducible dimensions, and high performance [1,2]. Therefore, PM steels have been extensively used for fabricating structural components in the automobile and mechanical fields [3]. Iron powder and its components are very important PM materials that are widely used in the automotive, machinery, electrical appliances, and other fields. However, the mechanical properties of PM sintered components are limited by the forming method and are inferior to those of ingot steels due to inherent inner porosity, which seriously degrades the properties, especially ductility and toughness [4,5]. Thus, improving the mechanical properties of PM components is a continuously pursued goal. 

Typically, high-performance PM components can be obtained through two main approaches: density enhancement and alloying. The method most used to obtain high-density PM components is to improve the pressing pressure to obtain high green and sintering densities. The pressing pressure for Fe-based PM components is 500–800 MPa [6]. Excessive pressing pressure leads to compaction die wear, mold damage, high equipment investment, and high production costs. Increasing the sintering temperature is another feasible method for obtaining high-density PM steels. The burdening temperature of the belt furnace for the industrial production of PM steels is 1120–1180 °C, and an excessive sintering temperature leads to equipment damage and an unacceptable increase in production costs. Other PM-forming technologies, such as double-press/double-sintering, surface Cu infiltration, powder sintering-forging, and warm compacting, can be used to produce high-density and high-performance PM steels. However, these techniques inevitably lead to high production costs and negative effects on mass production. 

On the other hand, alloying elements commonly include C, Cu, Mo, Ni, Cr, Mn, B, and rare-earth [7,8,9,10,11]. Specifically, C is the most effective and cheapest alloying element that can be used to modify the microstructure and improve the properties of PM components, and the microstructure, sintering temperature, and heat treatment technology of PM components are significantly affected by the C content [12,13,14,15]. Carbon functions as a solid lubricant to reduce the friction and wear of dies and improves the tensile strength of PM steels [16]. Copper does not fully dissolve in Fe, but its low melting point promotes the densification rate and density of PM components to improve their performance, while also affecting and controlling the dimensional accuracy of the PM components [17]. Molybdenum is seldom added to Fe powder in the form of a metallic powder because of the low diffusion velocity of Mo atoms at standard sintering temperatures. Previous studies have confirmed that the Mo diffusion velocity and distance were not adequate even when PM components were sintered at 1200 °C or higher for a long time [11,18]. Mo increases the hardness, tensile strength, and wear resistance of PM components by improving their hardenability [19,20,21]. Therefore, a series of Fe-*x*Mo (*x* = 0.5, 1.0, 1.5, respectively) pre-alloyed powders have been researched and developed to add Mo to Fe or Fe-based alloys to guarantee a homogenous distribution, short diffusion distance, and complete functions. Nickel is an austenitic enhancer that dissolves in Fe to form a continuous Fe-Ni solid solution, improving the tensile strength and toughness of PM components [22,23]. 

Other metallic elements, Cr and Mn, have attracted the interest of many researchers. Metallic Cr powder is seldom utilized in Fe-based PM components owing to its high affinity for O. Therefore, it is often atomized using high-pressure water or gas to obtain Fe-Cr pre-alloyed powders. Numerous studies have confirmed that Cr additions improve the wear resistance of PM components, by using appropriate sintering and surface treatment techniques [24,25,26]. The compressibility of Cr-containing powders is clearly lower than that of Fe and blended Fe-Cr hybrid powders. Manganese is also used in Fe and Fe-based PM components and Mn possesses similar characteristics to Cr. Manganese, which has been adopted as a cheap alloying element to reduce production costs, and Mn-containing PM components exhibit comparable performance as components with other alloying elements [27]. With the continuously increasing price of Cu, Mo, Ni, and other metals, Mn has become a competitive alloying element in PM components because of its low price. Moreover, rare-earth elements and their compounds are added to Fe and Fe-based alloys to improve the mechanical properties of PM components or other high-performance PM materials [28,29,30]. However, only small amounts of rare-earth elements are added to the Fe system because of their high cost and excessive rare-earth contents may cause severe deterioration in performance [31]. 

It is important to note that the compressibility of Fe powder is decreased by the addition of all alloying elements, no matter whether they are added as metallic powder or pre-alloyed powder. Specifically, the addition of Cr and Mn has a greater negative impact on compressibility than other elements. 

Boron is an active sintering element in Fe-based PM parts, but its industrial use is limited due to processing challenges. Nevertheless, researchers have explored the benefits of adding low amounts of B to PM steels and wrought materials. Unlike Cu and P, B has extremely low solubility in Fe, and the formation of the Fe-Fe_2_B eutectic at 1177 °C promotes the mass transport and densification rate of PM steels [32]. Thereby, boron can enhance the densification of PM steels by liquid phase sintering [33,34]. However, excessive B additions cause embrittling, owing to the brittleness of the (Fe,X)_2_B boride network formed during sintering [35]. Although boron has been frequently studied in the field of PM steels, few studies have yet systematically investigated the optimized B additions regarding the microstructure and mechanical properties of PM steel components. Therefore, this study comprehensively investigates the effects of B additions on the microstructural evolution and performance of Fe-Mo-Cu-Ni-C PM steels. 

## 2. Methods

Pre-alloyed powder D_1_, provided by Shandong Luyin New Material Technology Co., Ltd., China, and natural graphite powder were used in this study. Table 1 lists the chemical composition of the pre-alloyed powder D_1_. The average particle sizes of the pre-alloyed powder D_1_ and graphite were 100 mesh and 30 μm, respectively. Boron was added to the system in the form of FeB80 alloy powder with an average particle size of 30–40 μm, and the B contents in the system were 0, 0.1, 0.2, 0.3, 0.4, and 0.5 (wt.%). Table 2 lists the chemical composition of the specimens. Furthermore, 0.7% micro-powder wax was added to the powders as a lubricant. 

The specimens were compacted using a single application of 700 MPa at room temperature. The geometric size of the specimens prepared for the hardness and microstructural analyses was 10 × 10 × 12 mm^3^, and the 10 × 10 mm^2^ head face was prepared according to JB/T 2798–1999 national standards [36]. The hardness of the specimens was measured using an HR-150B Rockwell hardness tester (Jinan Huayin, China), and the recorded value was the average of five measurements at different positions on the test surface. The microstructures of the as-sintered and heat-treated steels were observed using an optical microscope. The steel specimens for the impact and tensile tests were prepared according to the ASTM E23 [37] and ISO 2740 standards [38], and the geometric dimensions of the specimens for the impact test were 10 × 10 × 55 mm^3^. All the specimens were vacuum sintered at 1140 °C for 40 min and cooled in the furnace. 

The as-sintered specimens were heated to 860 °C for 30 min while maintaining a C potential of 0.7% in the furnace to compensate for C loss, quenched in oil for 25 min at 55 °C, and then tempered at 180 °C in an annealing furnace for 240 min to reduce the quenching stress and extrude the retained quenching medium out of the specimens. Subsequently, the specimens were processed for testing. 

The densities of the specimens with different B contents were measured using the Archimedes method. The tensile strength was measured using a universal material testing machine (Kexin WDW-100, Changchun, China) with a crosshead velocity of 0.5 mm/min. IM testing was performed using an impact-testing machine (JBW-150, Jinan, China). The transmission electron microscope (TEM) observation was performed on TEM instruments (FEI Tecnai G2 F20, Hillsboro, OR, USA) with a 200 KV accelerating voltage to identify the borides. The wear resistance of the sintered steels was tested using an friction wear testing machine (Zhengli M-2000A, Zhangjiakou, China), and the loaded pressures on the ring-block friction counterparts were 100 and 200 N. The test process lasted 10 min, and the rotational speed of the coupled ring was 400 rpm (approximately 0.84 m/s). In general, all the density and mechanical tests were averaged at least three times. The worn wear surface was observed using SEM. The equilibrium phase diagrams were calculated using Pandat 2022 using the Fe-base alloy database. 

## 3. Results and Discussion

### 3.1. Sintering Technology and Densities

**Figure 1:**
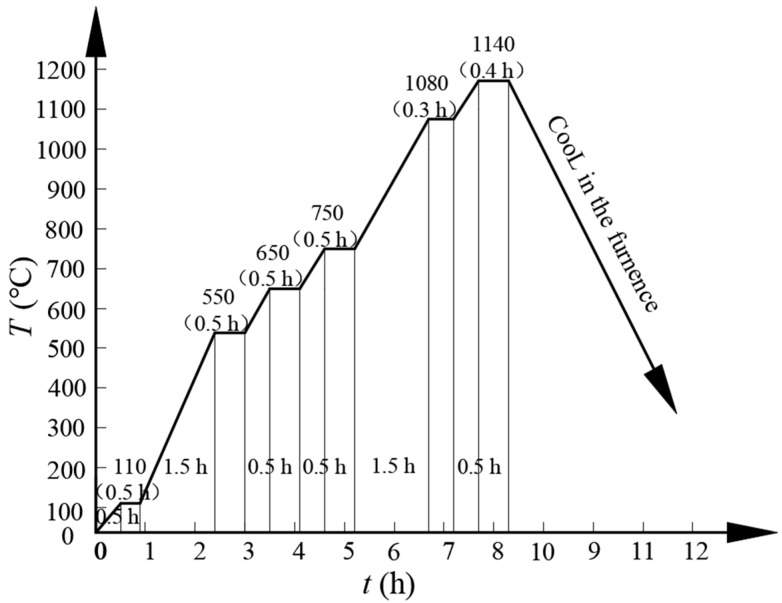
Master sintering curve of the steels used in this study.

**Figure 2:**
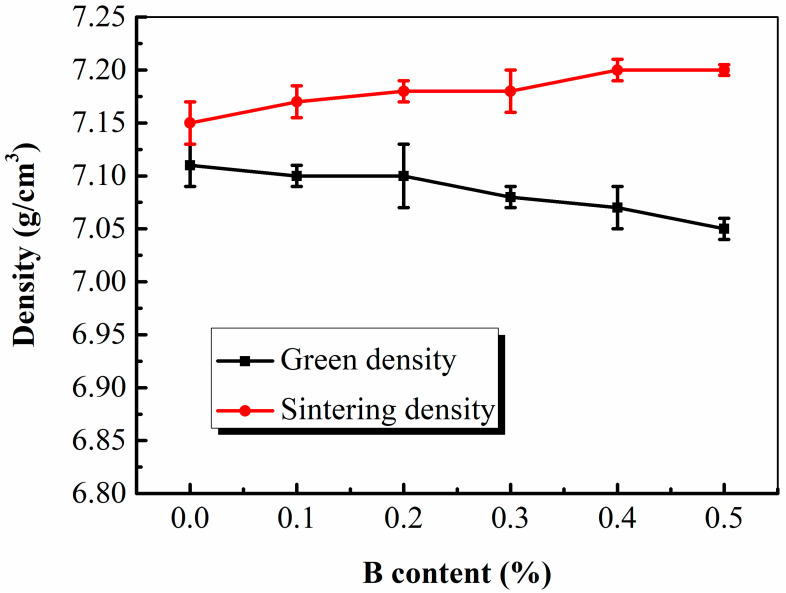
Effect of B content on the green and sintering densities of the specimens.

Figure 1 shows the master sintering curves of the steels employed in this study. Figure 2 shows the sintering and green densities of the specimens. Considering the melting point of Cu, the element, an insulating stage was set at 1080 °C for 18 min. The aim was to promote Cu liquid infiltration into the Fe particles to accelerate mass transportation. Concurrently, low melting point eutectic Fe-B liquid possibly occurred due to the fine and active Fe-B particles, even though the temperature did not reach the theoretical formation temperature of the Fe-B eutectic liquid (approximately 1177 °C under normal pressure conditions). Moreover, vacuum sintering implies a higher temperature than conventional atmosphere sintering in an N_2_ + H_2_ atmosphere. Once the liquid appeared during the sintering process, mass transportation and reactions rapidly occurred and were complicated. In contrast, an excessively accelerated temperature increase and a higher temperature led to volatilization owing to the high saturated vapor pressure of B. Additionally, the B-containing eutectic liquid rapidly flowed and tended to accumulate in the areas near the surface of the specimen, resulting in element segregation and the deteriorated performance of the steels. 

Figure 2 shows that the green density of the specimens slightly decreased with an increasing B content because the B additions and Fe-B hard particles remarkably reduced the compressibility of the mixed powders. However, the sintering density of the specimens increased with an increasing B content. The green density of the specimen without B additions was 7.11 g/cm^3^ and that of the specimen with a B content of 0.5% was 7.07 g/cm^3^. Vacuum sintering at 1140 °C for 24 min increased the densities of the PM steel specimens by approximately 0.04–0.13 g/cm^3^. Additionally, the maximum sintering density of the specimens with 0.4 and 0.5% B was 7.2 g/cm^3^. Although a high B content promoted the densification rate and density of the PM components, it was critical to control the dimensional tolerances owing to the shrinkage of B-containing components. The addition of B decreased the eutectic temperature of the PM steels, causing the liquid phase to appear at a lower temperature, promoting the densification of the PM steels. In this study, the vacuum sintering process resulted in a higher sintering temperature, as compared to conventional and economical sintering conditions (that is, sintering in a network belt furnace at 1120 °C for 40 min in a 90 vol.% N_2_-10 vol.% H_2_ mixed atmosphere and with an appropriate (0.5–1.0 °C/s) cooling rate). Therefore, vacuum sintering accelerated the tendency of the liquid phase to manifest at a lower temperature and the densification rate of the PM steels. 

The high density of the sintered specimens was also attributed to the excellent compressibility of the pre-alloyed powder D_1_, which was prepared using pre-alloyed Fe-0.5Mo powder bonded with Ni and Cu powders on a Fe powder surface. The Mo content of the pre-alloyed Fe-0.5Mo powder was 0.5%, which has a very slight negative effect on the compressibility. The microhardnesses of Ni and Cu powders are lower than that of the Fe-0.5Mo pre-alloyed powder; therefore, the Fe-0.5Mo pre-alloyed powder still exhibited favorable compressibility despite the alloying elements. 

### 3.2. Microstructural Characteristics of the Sintered Steels

**Figure 3:**
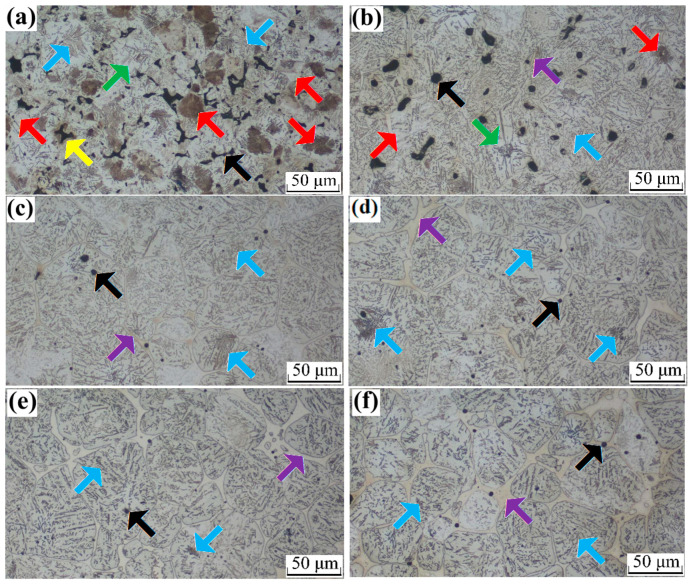
Optical images of the microstructure of the sintered specimens with different B contents of (**a**) 0, (**b**) 0.1, (**c**) 0.2, (**d**) 0.3, (**e**) 0.4, and (**f**) 0.5%, wherein the black arrow indicates the angular or irregular pores, the purple arrow shows net-like B eutectic network, the green arrow indicates Ni-rich austenite surrounding the surface of the base powder particles, the red arrow indicates ferrite phase, and the yellow arrow in shows a Cu-rich area.

Figure 3 illustrates the microstructures of the sintered specimens after etching with nital. The results suggest that the microstructure of the sintered steels is more complex than that of the wrought steel with the same composition. The microstructure of PM steel is strongly influenced by various factors, such as the green density, type and number of alloying additives, type and size of the initial powders, and sintering processes (temperature and time), particularly the cooling conditions and heat treatment processes. As a result, the microstructure of different powders or the microstructure of different regions varies, due to the processing method employed.

As shown in Figure 3a (specimen 1^#^ without B additions), the microstructure of the sintered steel primarily consisted of pearlite and ferrite, Ni-rich austenite, Cu-rich pearlite, and pores. The red and blue arrows in Figure 3a indicate the ferrite and pearlite phases of the base powder, respectively; the color of the ferrite phase was darker than that of the pearlite phase. The ferrite phase (red arrows indicated) formed because no or a very small amount of graphitic C diffused and dissolved in these regions, and Mo is a ferrite stabilizer [39]. Although the pearlite phase (blue arrows indicated) formed because sufficient C diffused and dissolved in these regions, it was noted that bainite could not form during vacuum sintering under a low cooling rate, which led to the absence of the martensite phase. The green arrow indicates Ni-rich austenite surrounding the surface of the base powder particles. The Ni-rich austenite areas and Cu-rich pearlite areas were also located around the pores, which was different from the results reported in previous references [40,41], because high sintering temperature and slow cooling velocity in the furnace promoted Ni diffusion. The yellow arrow in Figure 3a shows a Cu-rich area around a pore, which is possibly the pearlite phase rather than martensite owing to the sintering process and sufficient C content accumulated here by the Cu liquid during sintering. The black arrow in Figure 3a indicates the inherent pores in PM steel, the shape of which was angular or irregular and significantly influenced the performance. The shape of the pores is closely related to the green density, initial powders, alloying elements, sintering processes, and other factors.

Figure 3b–f shows the microstructures of the sintered steels with B additions of 0.1–0.5%, respectively. The microstructure of the sintered steels with B additions clearly evolved and was different from that of the PM steel without B additions, and consisted of pearlite, ferrite, Ni-rich austenite, Cu-rich pearlite, B eutectic phase, and pores. The quantity of the pearlite phase in the sintered steels increased with an increasing B content, because the B eutectic appeared at a lower temperature and promoted the flow of liquid Cu along the Fe particles. Carbon, Ni, and Cu are favorable for redistribution and alloying; therefore, the quantity of pearlite increased and the quantity of ferrite decreased, and the ferrite phase gradually disappeared with a further increase in the B content. Some studies have classified pearlite as coarse pearlite or fine pearlite in different positions of the material [9,19]; however, it was difficult to distinguish between them in this study. The Ni-rich areas decreased with an increasing B addition because the liquid (melted Cu and B eutectic) promoted Ni redistribution. The Cu-rich area also decreased and then disappeared with an increasing B addition. The purple arrow indicates the B eutectic along the Fe particles, which promotes densification and alloying element redistribution; however, B eutectic nets appeared when the B content exceeded 0.2%, which significantly deteriorated the performance of the sintered steels by imparting brittleness and low ductility. The number and shape of the pores is another significant microstructural evolution in the sintered steels with B additions. The number of pores decreased and the shape of the pores became rounder with an increasing B content. The pores almost disappeared when the B content exceeded 0.2%. 

**Figure 4:**
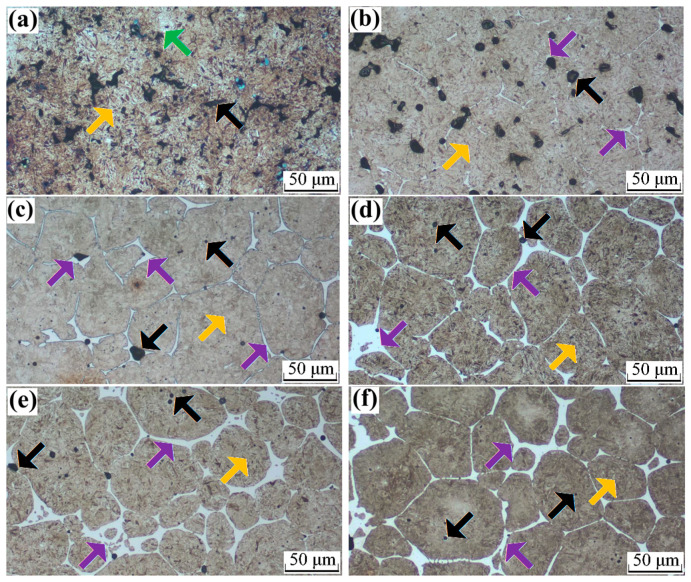
Etched microstructure of the sintered specimens with different B contents of (**a**) 0, (**b**) 0.1, (**c**) 0.2, (**d**) 0.3, (**e**) 0.4, and (**f**) 0.5%, wherein the black arrow indicates the angular or irregular pores, the purple arrow shows net-like B eutectic network, the green arrow indicates Ni-rich austenite surrounding the surface of the base powder particles, the red arrow indicates ferrite phase, and the yellow arrow in shows a Cu-rich area.

Figure 4a–f shows the heat-treated microstructures of the sintered steels with different B contents of 0, 0.1, 0.2, 0.3, 0.4, and 0.5%, respectively. Figure 4a shows that the main constituent of the microstructure of the B-free steel was martensite, as indicated by the orange arrow. However, small Ni-rich areas, marked by a green arrow, were observed, confirming the presence of retained austenite or “isolated austenite islands”. The black arrow indicates the angular or irregular pores, similar to that described for the as-sintered steels, which significantly influenced the properties of the sintered steel. Figure 4b–f shows the microstructures of the sintered B-containing steels, which show that the microstructure of the heat-treated B-containing steels was predominantly composed of martensite, which coarsened with an increasing B content because the liquid B eutectic promoted particle bonding and coalescence. Retained austenite was hardly observed and/or was overlapped by the liquid B eutectic and alloying element redistribution and completely disappeared with a further increase in the B content. The net-like B eutectic network (indicated by the purple arrows), which deteriorated the performance of the sintered steels, leading to low ductility and brittleness, was clearly visible when the B content exceeded 0.2%. The pores of the sintered steel after heat treatment were round, and the pore size decreased with an increasing B content. The low melting point of the liquid B eutectic promoted densification and changed the pore shape and content.

**Figure 5:**
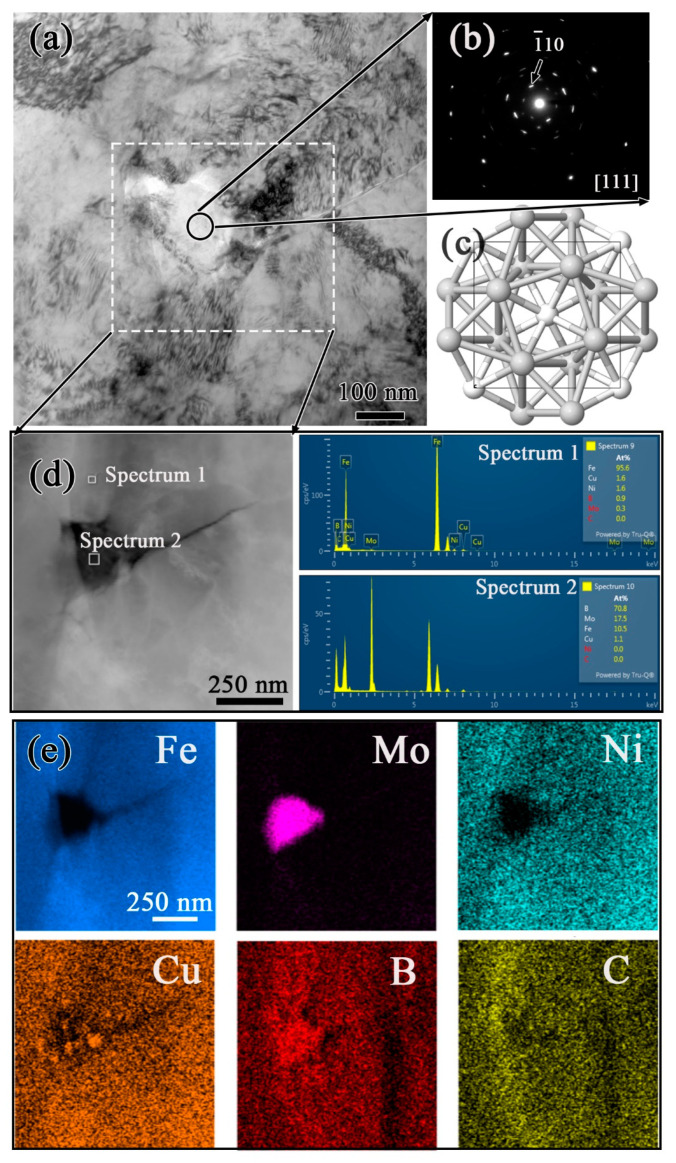
Transmission electron microscope (TEM) observation results of Fe-Mo-Cu-Ni-C sintered steel with 0.5% B: (**a**) micrograph of precipitate; (**b**) [111] selected area electron diffraction (SAED) pattern; (**c**) ball-and-stick model for visualizing C16- type Mo_2_B structures with the space group of I4/mcm; (**d**) enlarged micrograph showing (Fe, Mo)_2_B precipitate and EDS energy spectrums of selected regions; (**e**) elemental mapping of the selected region.

Basically, the microstructure of sintered steels is closely related to the green density, chemical composition, type of initial powders, sintering processes and, thereafter, heat treatment processes, alloying elements, and inner defects. The preparation process of D_1_ powder was different from that of premixed or pre-alloyed powders, and it was determined to be a type of hybrid powder as it was prepared using Ni and Cu metallic powders bonded with Fe-0.5Mo alloyed powder. Therefore, the microstructure of the sintering steel with D_1_ powder was different from that of premixed and pre-alloyed sintered steels. Furthermore, transmission electron microscope (TEM) analyses were applied to investigate the existing formation of B element. As illustrated in Figure 5, the EDS energy spectrum and element mapping indicated that the Fe-, Mo-, and B- rich phases were formed; the [111] selected area electron diffraction (SAED) pattern conformed to the precipitation of C16- type M_2_B structures with the space group of I4/mcm, which is depicted in Figure 5c.

**Figure 6:**
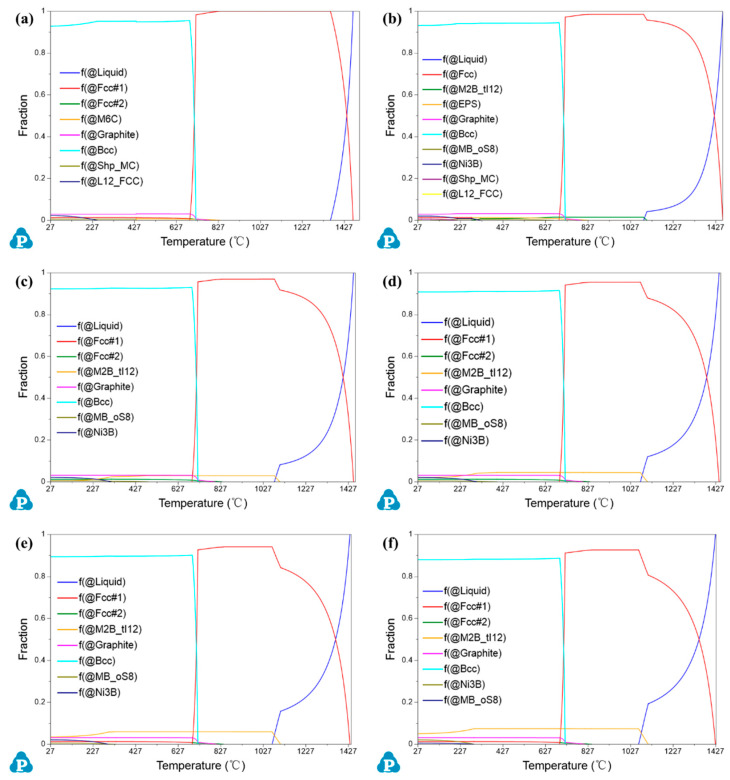
The equilibrium phase diagram of Fe-Mo-Cu-Ni-C steel with different B additions: (**a**) 0, (**b**) 0.1%, (**c**) 0.2%, (**d**) 0.3%, (**e**) 0.4%, and (**f**) 0.5%.

The equilibrium phase diagram conformed to the formation of M_2_B in Fe-Mo-Cu-Ni-C steel with B additions. As shown in Figure 6, with the increase in B addition, the content of M_2_B-type boride with precipitation at 1100 °C is increased. In addition, the addition of B can also induce the precipitation of MB and Ni_3_B phases, while their precipitation temperature and content are too low, hence these phases are restrained and can hardly be observed in the B-added samples.

### 3.3. Mechanical Properties

**Figure 7:**
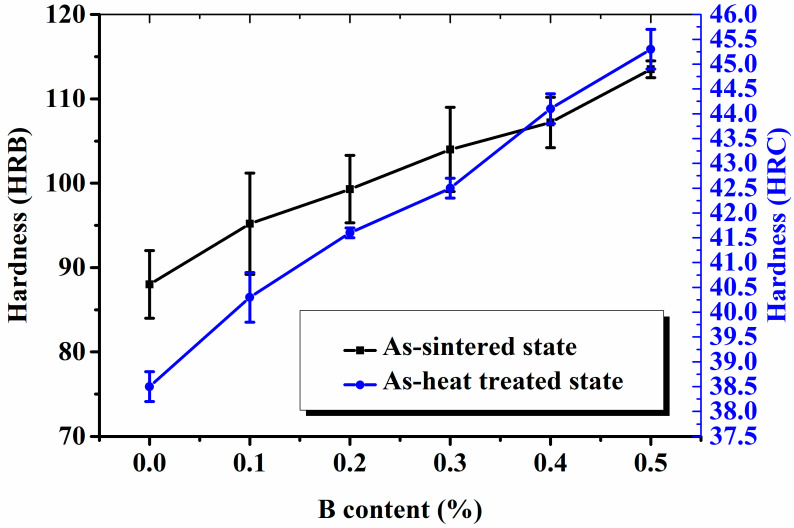
Hardness of the specimens in the as-sintered and heat-treated states.

The mechanical properties, hardness, tensile strength, and IM of the sintered steels were comprehensively investigated. The hardness of the as-sintered and heat-treated specimens was measured using a Rockwell hardness tester, and the results are shown in Figure 7. The hardness of the specimens monotonously increased with an increasing B content, and the hardness of the as-sintered and heat-treated specimen 6^#^ (B content of 0.5%) reached maximum values of HRB 113.5 and HRC 45.3, respectively. The hardness of specimen 2^#^ (B content of 0.1%), as compared to that of B-free specimen 1^#^, noticeably increased; subsequently, the rate of increase in the hardness slightly decelerated. The B additions decreased the eutectic melting point and promoted densification of the sintered steels; thus, the hardness of the specimens increased with an increasing B content. Another reason for the increase is that B reacts with Mo to form multiple B-containing hard particles, which strengthen and harden the steels; however, further research is needed to confirm this.

**Figure 8:**
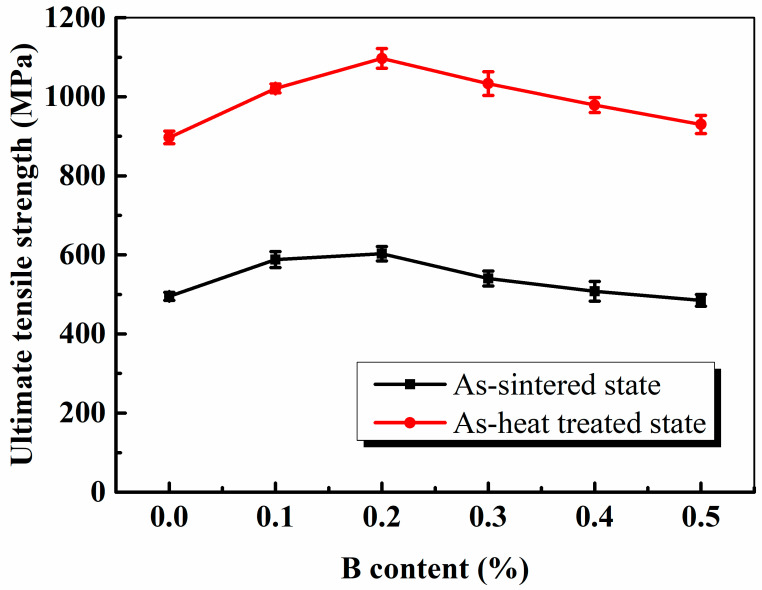
Effect of the B content on the tensile strength of the specimens in the as-sintered and heat-treated states.

It is known that the static properties of sintered steels are dependent on their total porosity, chemical composition, and microstructure [12]. Figure 8 shows the ultimate tensile strengths (UTS) of the sintered and heat-treated steels. The ultimate tensile strength of the steels in the as-sintered state first increased and then decreased with an increasing B content, reaching a maximum value of 603 MPa at a B content of 0.2%. The UTS of the as-heat-treated steels showed the same tendency as that of the as-sintered steels, and the UTS of specimen 3^#^ reached a maximum value of 1097 MPa when the B content was 0.2%. Boron decreases the eutectic temperature of the steel, and the liquid phase appears at a lower temperature to promote the densification rate and modify the pore shape and pore type of the PM steels; thus, the tensile strength of the sintered steels improved with increasing B content. In contrast, the liquid B eutectic promoted the reaction of B and Mo in the inner Fe particles to strengthen the steel, and the B additions significantly modified the microstructure of the steel. However, more liquid B eutectic appeared earlier with an increasing B content, and decreased the sintering neck bonding strength of the Fe particles, which deteriorated the properties of the PM steels. Therefore, the tensile strength of the sintered steels decreased with further increasing B content.

**Figure 9:**
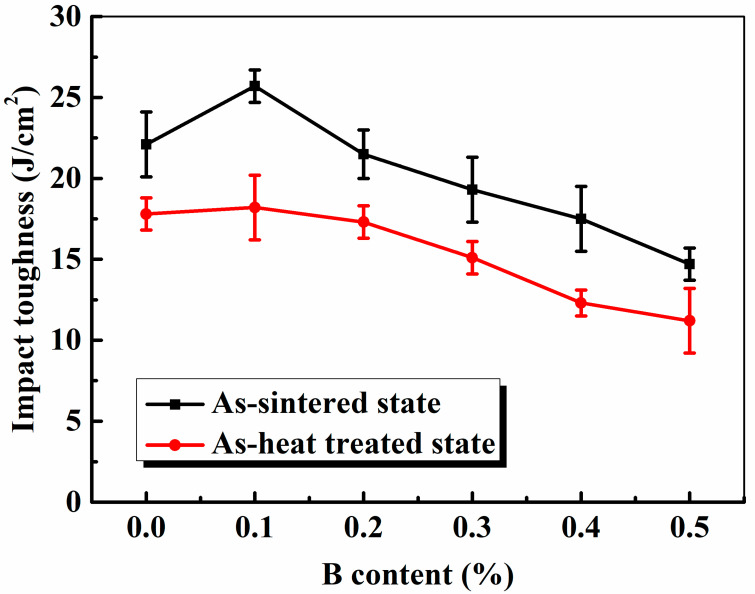
Effect of the B content on the IM of the as-sintered and heat-treated specimens.

Figure 9 shows the IM values of the as-sintered and heat-treated steels. Generally, the IM of PM steels is closely related to their density, porosity, chemical composition, alloying elements, and microstructures. The IM increases with the improved density and decreased porosity of sintered steels. The IM of the as-sintered PM steels was attributed to the combination of the excellent plasticity and ductility of ferrite and the good strength, hardness, and appropriate plasticity and ductility of pearlite. The maximum IM of the as-heat-treated PM steels with a 0.1% B content was 25.7 J/cm^2^, which abruptly decreased with a further increase in the B content. The increase in the IM of the steels with B additions was attributed to the densification rate and increased density of the B additive, leading to the appearance of the liquid phase at a lower temperature. With a continuing increase in the B content, a net-like B eutectic formed, which reduced the bonding strength between the Fe particles; thus, the IM abruptly decreased owing to the brittleness of excessive B additions in PM steels. In this study, the dominant microstructure of the PM steels became hard and brittle martensite with Ni, Mo, and Cu dissolved in the matrix, and internal stress existed after heat treatment; therefore, the IM of the steels after heat treatment decreased compared to that of the as-sintered steels, and it was boosted by the B additive. The IM of the heat-treated steels with the 0.1% B addition was slightly higher than that of the steel without B additions because of the higher density and modified pore shape and pore type, which reduced the inner defects and stress.

**Figure 10:**
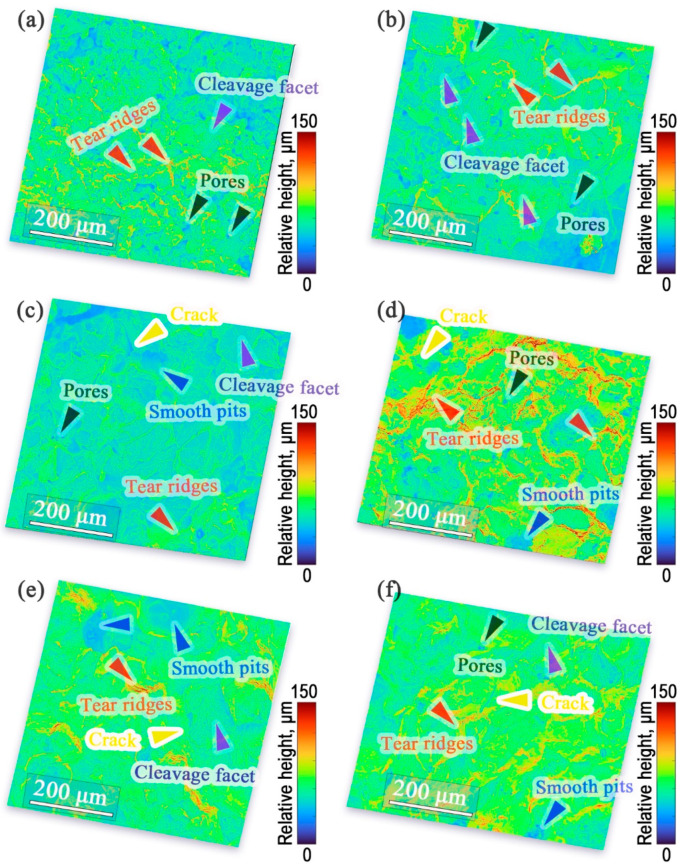
Impact fracture morphology of the sintered steels with different B contents: (**a**) 0, (**b**) 0.1%, (**c**) 0.2%, (**d**) 0.3%, (**e**) 0.4%, and (**f**) 0.5%.

Figure 10 illustrates the impact fracture morphology of the heat-treated PM steels with varying B content. The fracture mode of the steels was an intergranular and transgranular composite rupture. As shown in Figure 10a, the B-free steel exhibited an intergranular fracture, suggesting that the main failure mode was debonding between the Fe particles. This indicates that the bonding strength between the Fe particles in the PM components was always weak. Numerous dimples and tear ridges marked by red arrows were observed, but the extent of deformation was small. Additionally, some Fe particles were split off, leading to a transcrystalline rupture and the formation of cleavage facets. 

The fracture morphology of the B-containing steels (Figure 10b–e) was different to that of the B-free steel, as shown in Figure 10a. The dimpled areas and quantity of tear ridges in the steel with a 0.1% B addition (Figure 10b) were larger and more obvious than those in the B-free steel (Figure 10a), which increased the impact energy of the steels and was consistent with the IM result. However, a greater transcrystalline rupture is observed in Figure 10b, which was due to the increased bonding strength between the Fe particles. Owing to the high ductility and strength of the Fe particles, the transcrystalline rupture of Fe particles consumed considerable energy to improve the IM of PM steel, and perhaps the (Fe,Mo)(B,C) hard particles formed inside the Fe particles increased this tendency. In the samples with B additions over 0.2%, the deformation level of the dimples and tear ridges became slight and incomplete, and the transcrystalline rupture of Fe particles is scarcely observed in Figure 10d,e, which significantly decreased the IM of the steels, confirming that the brittleness of the PM steels increased and the IM decreased with an increasing B content. To improve the comprehensive properties of PM steels, the B content must be carefully optimized. This study found that the B content for Fe-Mo-Ni-Cu-C steels prepared with D_1_ powders should not exceed 0.2% (wt.%). The black arrows in Figure 10 indicate that the pores nearly disappeared. Smooth pits were observed, which indicated that the Fe particles debonded with particles (indicated by blue arrows), and that cracks were found (indicated by yellow arrows) with an increasing B content, demonstrating the brittleness and low ductility of B-containing sintered steels.

### 3.4. Wear Behavior and Mechanism

**Figure 11:**
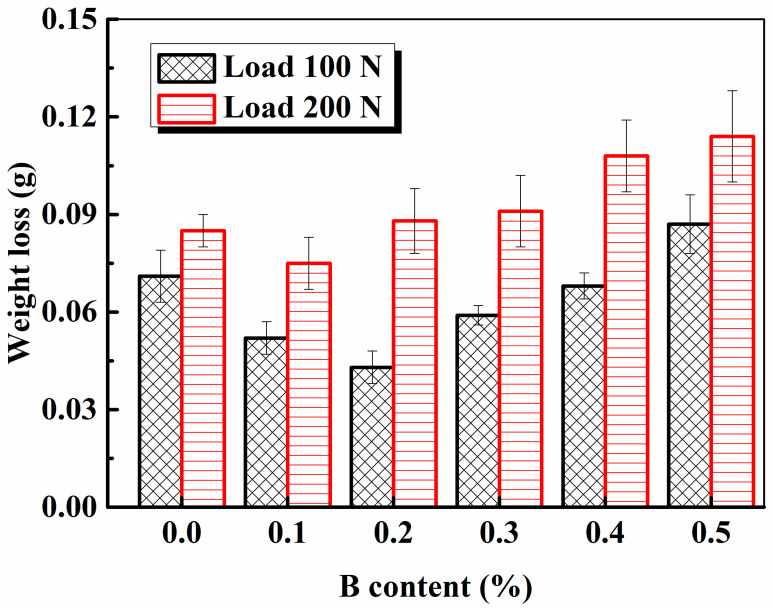
Effect of the B content on the wear resistance of the sintered steels under different load conditions.

Figure 11 shows the weight losses of the sintered steels after wear tests.

**Figure 12:**
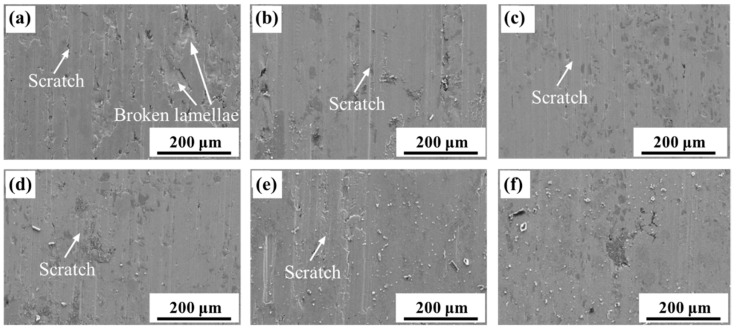
Frictional morphology of the steels with different B contents under low loads (100 N): (**a**) 0, (**b**) 0.1, (**c**) 0.2, (**d**) 0.3, (**e**) 0.4, and (**f**) 0.5%.

The weight loss of the sintered steels first decreased and then increased, regardless of the applied load. Under low load conditions, the weight loss of specimen 2^#^ (0.2% B addition) was the lowest. Figure 12 shows the wear surface morphology of the steels under low load conditions. It is evident that the B-free specimen 1^#^ exhibited significant breaking of lamellae from its surface, while the scratches observed on the surfaces of B-containing steels were relatively minor. This confirmed that the damage mechanism of the PM steels under low load conditions was adhesion/triboxidation, which is the typical wear mechanism in dry sliding [19].

**Figure 13:**
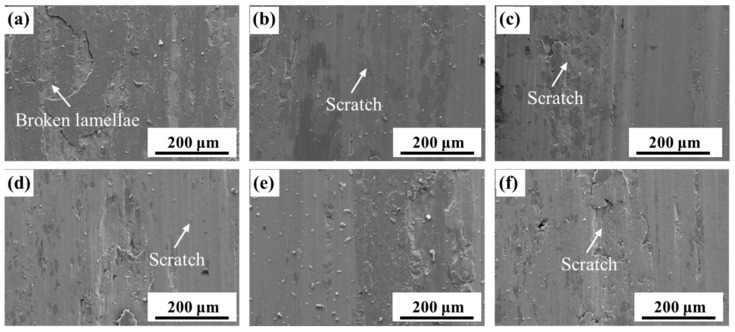
Frictional morphology of the steels with different B contents under a high load (200 N): (**a**) 0, (**b**) 0.1, (**c**) 0.2, (**d**) 0.3, (**e**) 0.4, and (**f**) 0.5%.

Figure 11 also indicates that the heat-treated steel with a B content of 0.1% (specimen 2^#^) exhibited the best wear resistance under high load conditions, as compared to the other steels. The wear resistance of the steels decreased with an increasing B content. Additionally, the wear resistance of the steels with a B content over 0.2% (specimens 3^#^–6^#^) was inferior to that of the B-free steel (specimen 1^#^). Figure 13 shows the worn surface morphology of the sintered steels under high load conditions. Larger pieces of lamellae were observed to have broken off from the surface, which indicated that microcracks occurred in the subsurface, propagated, and led to the production of metallic fragments, which is known as the “three body wear” phenomenon. Oxidation of the surface or subsurface during the wear test promotes this process. With the addition of B, the friction coefficient of the PM steels decreased owing to the formation of boronic oxide; however, the wear resistance of the steels with B contents of 0.2–0.5% (specimens 3^#^–6^#^) was still lower than that of the B-free steel (specimen 1^#^). With a further increase in the B content, external stress on the contact surface led to cracks initiating and propagating to form metallic fragments owing to the brittleness of the excess B additions; thus, abrasive wear also occurred. Therefore, the failure modes of the steels under high load conditions were abrasion wear, triboxidation, and adhesive wear.

## 4. Conclusions

Fe-0.5Mo-1.5Cu-1.75Ni-0.7C steels were manufactured with pre-alloyed powder D_1_ as the raw material and a 0.7% graphite addition using PM techniques to investigate the effects of different B additions on the microstructure and properties of Fe-Mo-Cu-Ni-*x*B-C sintered steels. The main conclusions were as follows:

(1) The microstructures of the sintered steels with and without the B additions consisted of pearlite, ferrite, Ni-rich austenite, Cu-rich pearlite, and pores; however, as the B content increased, the ferrite phase became smaller and even disappeared, and Ni and Cu elements became more homogeneous. After the heat treatment, the microstructure of the steels mainly consisted of martensite. The pores became rounder and the number of pores decreased and disappeared with an increasing B content.
(2) The C16-type Mo_2_B structures with the space group of I4/mcm were identified by transmission electron microscope (TEM) observation, and the equilibrium phase diagram calculation indicated that the B addition facilitated the formation of the M_2_B phase.
(3) The sintering density of the PM steels increased as the B content increased, and the hardness of the materials monotonously increased. The ultimate tensile strength of the heat-treated steels attained a maximum value of 1097 MPa with a B content of 0.2% and the IM of the steels attained a maximum value of 25.7 J/cm^2^ with a B content of 0.1%.
(4) The wear resistance of the steels initially increased and then decreased with an increasing B content. Under low load conditions, the 0.2% B steel exhibited excellent wear resistance due to the combination of good hardness and tensile strength, and the failure mode of the steel was mainly adhesion/triboxidation. Under high load conditions, the 0.1% B steel exhibited excellent wear resistance due to the combination of good hardness, strength, and excellent toughness, and the failure mode of the steel was abrasion wear, triboxidation, and adhesive wear.

## Figures and Tables

**Table 1:** Chemical composition of pre-alloyed powder D_1_ (wt.%).

Fe	Cu	Ni	C	Si	Mn	P	S	Mo	O
Bal.	1.45~1.52	1.70~1.75	≤0.015	≤0.10	≤0.35	≤0.016	≤0.025	0.45~0.50	≤0.25

**Table 2:** Nominal chemical composition of the sintered steels used in this study (wt.%).

Specimens	Fe	Mo	Cu	Ni	B	C	Wax
1^#^	Bal.	0.5	1.5	1.75	0	0.7	0.7
2^#^	Bal.	0.5	1.5	1.75	0.1	0.7	0.7
3^#^	Bal.	0.5	1.5	1.75	0.2	0.7	0.7
4^#^	Bal.	0.5	1.5	1.75	0.3	0.7	0.7
5^#^	Bal.	0.5	1.5	1.75	0.4	0.7	0.7
6^#^	Bal.	0.5	1.5	1.75	0.5	0.7	0.7

## Data Availability

The data presented in this study are available on request from the corresponding author.
